# Frequent inappropriate implantable cardioverter defibrillator therapy was determined to be dual atrioventricular nodal non-reentrant tachycardia: A case report

**DOI:** 10.1097/MD.0000000000025370

**Published:** 2021-04-09

**Authors:** Chengming Ma, Xiaomeng Yin, Yunlong Xia, Wenwen Li, Lianjun Gao, Shiyu Dai, Xiaohong Yu

**Affiliations:** Department of Cardiology, Institute of Cardiovascular Diseases, First Affiliated Hospital of Dalian Medical University, Dalian, China.

**Keywords:** dual atrioventricular node non-reentrant tachycardia, inappropriate implantable cardioverter defibrillator therapy, ischemic heart disease

## Abstract

**Rationale:**

Dual atrioventricular node non-reentrant tachycardia (DAVNNRT) is a rare arrhythmia. We present a case of inappropriate implantable cardioverter defibrillator (ICD) therapy caused by DAVNNRT. DAVNNRT is easily misdiagnosed as atrial fibrillation and is often identified as ventricular tachycardia (VT) by the supraventricular tachycardia-ventricular tachycardia (SVT-VT) discriminator of the ICD.

**Patient concerns:**

A 73-year-old man with ischemic heart disease (IHD) presented with palpitations accompanied by dyspnea and syncope. Frequent multifocal premature ventricular beats and non-sustained ventricular tachycardia were observed on ambulatory electrocardiography. The left ventricular ejection fraction decreased to 32%.

**Diagnosis:**

He was diagnosed with IHD, heart failure with reduced ejection fraction (HFrEF), and VT.

**Interventions:**

: Initially, the patient received a single-chamber ICD implantation for secondary prevention of sudden death. He then suffered from inappropriate anti-tachycardia pacing (ATP)/shock therapy many times after the procedure. DAVNNRT was confirmed in an electrophysiology study (EPS), and radiofrequency ablation of the slow pathway successfully terminated this tachycardia.

**Outcomes:**

No episode of inappropriate ICD therapy or tachycardia occurred during the follow-up.

**Lessons:**

In conclusion, it is essential to have a full understanding of DAVNNRT and eliminate slow pathways for patients with DAVNNRT and be prepared to implant an ICD.

## Abstract 2

Supplemental Digital Content is available in the text

## 1 Introduction

Implantation of an implantable cardiac defibrillator (ICD) is an effective method to protect against sudden cardiac death (SCD) in patients; however, inappropriate ICD therapy is common in the real world. Supraventricular tachycardia (SVT), such as atrial fibrillation, is a common cause. Here, we present the first case of inappropriate ICD therapy due to dual atrioventricular node non-reentrant tachycardia (DAVNNRT) in China. DAVNNRT is a rare type of SVT and is often identified as ventricular tachycardia by the supraventricular tachycardia-ventricular tachycardia (SVT-VT) discriminator of the ICD.

## 2 Case report

A 73-year-old man with ischemic heart disease presented with palpitations accompanied by dyspnea and dizziness for almost 1 year. Ambulatory electrocardiography showed frequent multifocal premature ventricular beats and non-sustained VTs. He suffered from syncope and received direct current cardioversion before he came to our center; unfortunately, the electrocardiogram (ECG) was recorded by an external defibrillator and was not preserved. He received emergency percutaneous coronary intervention (PCI) therapy for acute myocardial infarction and received drug-eluting stents in the left circumflex (LCX) artery in another hospital 3 years earlier. Physical examination showed no positive signs on admission. Transthoracic echocardiography revealed an ejection fraction of 32%, with a hypokinetic inferior and inferolateral wall. Hypersensitive troponin I (HsTnI) and brain natriuretic peptide (BNP) levels increased to 0.463 μg/L and 499.18 ng/L, respectively. Subsequently, the patient underwent repeat PCI therapy, and coronary angiography (CAG) showed 80% to 85% stenosis of the left anterior descending (LAD) artery as well as the LCX artery. Consequently, drug-eluting stents were placed in the LAD artery and balloon inflation was successfully performed in the LCX artery. Considering the concurrence of ischemic heart disease (IHD), heart failure with reduced ejection fraction (HFrEF), and syncope due to VT, a single-chamber implantable cardioverter defibrillator (ICD) (Iforia7 VR-T DX Biotronik SE&Co. KG, Berlin, Germany) was implanted for secondary prevention of sudden death. ICD settings were as follows: mode = VDI; VT1/VT2/VF-detection-rate = 167/180/200 beats per minute (BPM); detection/redetection counter = VT1:40/30, and VT2:16/14.

**Figure 1:**
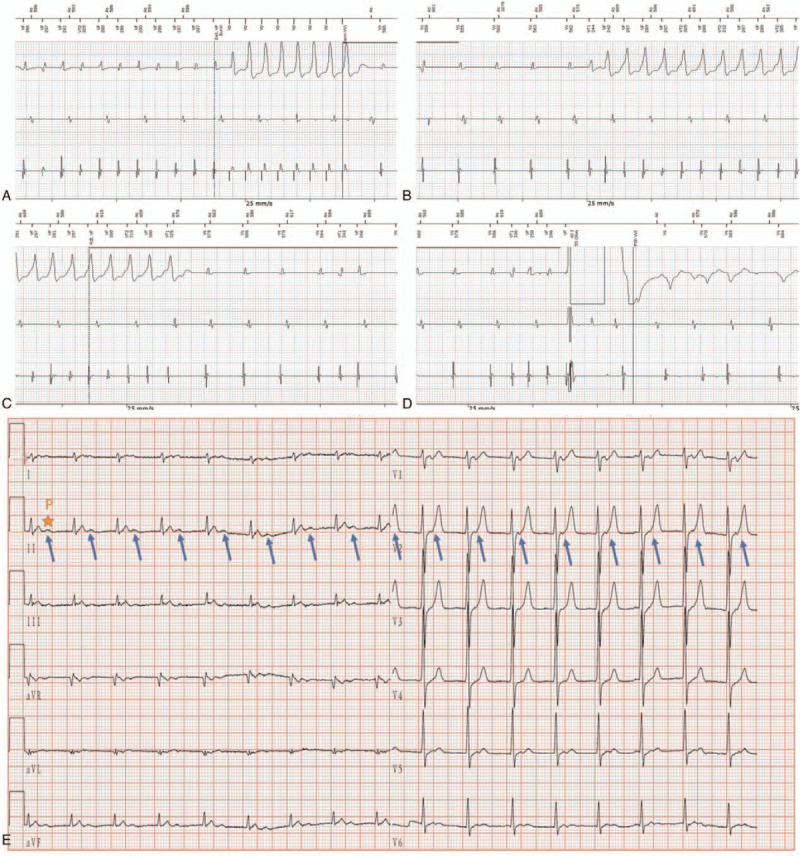
Continuous intracardiac electrogram recorded by ICD and surface ECG. (A) shows a tachycardia with 1:2 AV conduction. Burst stimulation by ICD terminated it. (B) and (C) recorded a cluster of widened QRS complexes and redetected the VF. ICD carried out a 40 J shock, as shown in (D). (E) shows a long PR interval.

**Figure 2:**
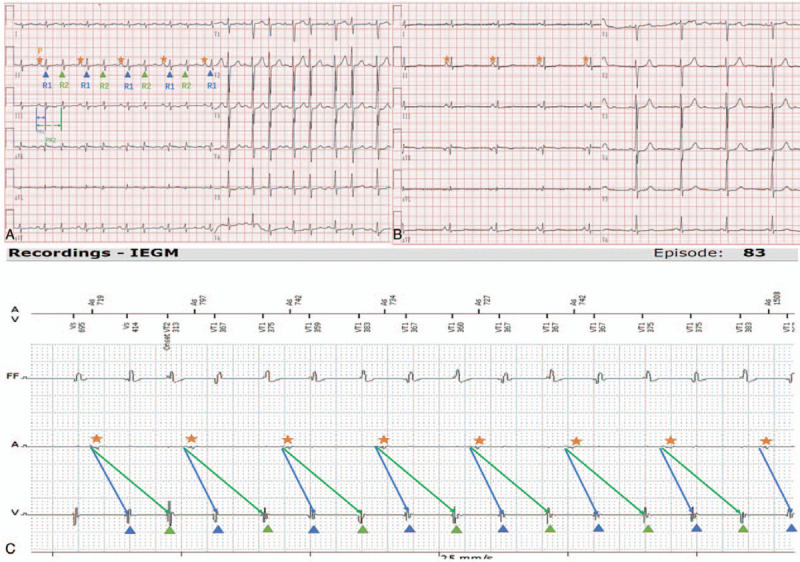
Surface electrocardiogram and IEGM from ICD programmer (25 mm/s, 10 mm/mV, 40 Hz). (A) shows a tachycardia with 1:2 AV conduction. One single P wave (marked 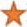) with 2 QRS complexes (marked 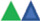) appeared regularly and alternately. P wave (marked 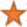) during sinus rhythm is shown in (B). The IEGM from the ICD in (C) demonstrates a clear 1:2 relationship between the atria and ventricles.

**Figure 3:**
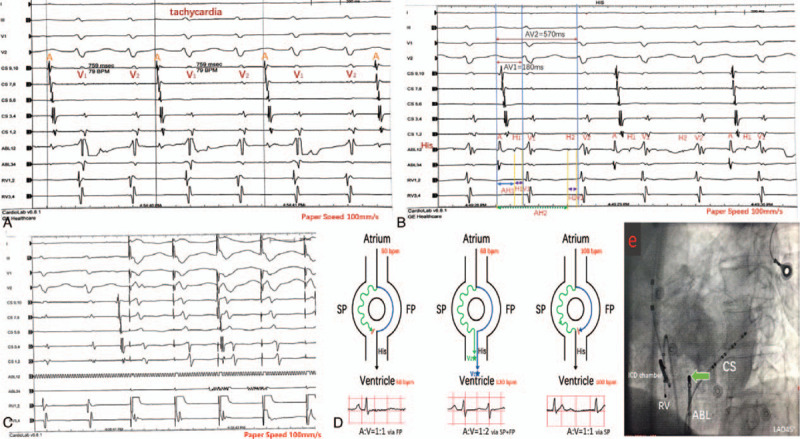
Intracardiac electrogram during EPS study. (A–C) shows surface ECG and IEGM during the tachycardia. (D) demonstrated the proposed mechanism of supraventricular beat conduction down the fast (left panel) and the slow pathway (right panel), respectively, and DAVNNRT (middle panel). (E) is a LAO 45° view of successful ablation (solid green arrow). AV = atrioventricular interval, AH = atrial-His bundle interval, HV = his-ventricular interval, SP = slow pathway, FP = fast pathway, LAO = left anterior oblique.

Unfortunately, he developed tachycardia. He underwent ICD therapy, including 173 rounds of anti-tachycardia pacing (ATP) and 5 rounds of shocks, in the 4 months following the ICD procedure (Fig. 1a−d). To avoid further inappropriate ICD therapy, we increased the VT1/VT2 detection rate and prolonged the VT detection time to avoid inappropriate ICD therapy. Additionally, amiodarone (600 mg per day) and metoprolol (23.75 mg per day) were prescribed, but they seemed to have no effect. The AV interval of the first 4 A and V waves in Figure 1b was approximately 570 ms, as shown in Figure 1e, which had a long PR interval. We found several series of narrow QRS complexes, which were automatically diagnosed as VT/ventricular fibrillation (VF) by the device, and we performed ATP therapy to terminate the VF (Fig. 1a). However, no ECG was recorded until the third admission. The ECG on the third admission (Fig. 2a) showed a narrow QRS complex tachycardia (NCT). The P waves in Figure 2a had the same morphology and axis as the P waves shown in Figure 2b, suggesting they were of sinus node origin, and each P wave occurred regularly with 2 QRS complexes following. It was noted that the R1R2 and R2R1 intervals showed the same regular pattern throughout the tracing, and the PR interval (PR1 and PR2) alternated between short and long intervals. Moreover, the second QRS presented with a relatively constant coupling interval after the short PR conducted beat. The morphology of the 2 QRS complexes was slightly different. This regularity indicated that the 2 related QRS complexes were generated by 1 given P wave, and the relationship was illustrated more clearly in an intracardiac electrogram (IEGM) from the ICD (Fig. 2c). The patient received EPS, and no VT was induced. The NCT mentioned above had a sudden and spontaneous onset with no programmed stimulation (Fig. 3). The surface ECG shown at the top of Figures 3a and b is the same as that in Figure 2a. A CS 9, 10 catheter clearly recorded 1 high-amplitude atrial potential (marked A), along with 2 low-amplitude ventricular potentials (marked V1V2). As shown in Figure 3b, the radiofrequency ablation catheter (ABL) was positioned at the His bundle to mark his potential. We found 1 a wave followed by 2 H-V waves: H1-V1 and H2-V2. The AH1 and AH2 intervals were 120 ms and 510 ms, respectively. The latter had a tremendous prolongation compared with the former. The HV interval was fixed, and both H1V1 and H2V2 intervals were 60 ms. Atrial burst stimulation (cycle length = 340 ms) can terminate tachycardia. Atrial S1S2 programmed stimulation (450/400 ms, −10 ms) was applied until the AVN refractory period was reached (450/360 ms). No “jump” phenomena were observed. Right ventricular apex pacing (S_1_S_1_) with multiple cycle lengths showed no V-A retrograde conduction (Fig. 3c). The difference between AH1 and AH2 established that atrial excitation conducts down to the ventricle with 2 divided pathways of the AVN, which had pronounced differences in conduction velocities when compared with each other. Retrograde conduction between the ventricle and atrium was absent. We called this type of tachycardia, dual atrioventricular node non-reentrant tachycardia (DAVNNRT). The middle panel of Figure 3d shows the electrophysiological mechanism of this tachycardia. Atrial beats being conducted to the ventricle along a slow pathway might explain the long AV and PR intervals shown in Fig. 1b and e because the AV intervals had approximately the same length as the AV_2_ intervals, as indicated in the EPS (Fig. 3b). Radiofrequency ablation of the slow pathway (Fig. 3e) terminated this tachycardia successfully, and the double ventricular response disappeared. We performed the EPS experiment again: ventricular pacing showed no V-A retrograde conduction, and programmed stimulation at the high right atrium as well as the CS7, 8 catheter could not induce tachycardia, which was the same as an intravenous drip of isoprenaline. No tachycardia occurred spontaneously.

There was no inappropriate ICD therapy or tachycardia during follow-up, and the IEGM from the ICD showed normal AV intervals (Supplemental file, http://links.lww.com/MD2/A47, and Supplementary data for reviewer http://links.lww.com/MD2/A48).

## 3 Discussion and conclusion

Here, we report a case of inappropriate ICD therapy due to DAVNNRT, which was undiagnosed before ICD implantation. ICDs are effective in protecting patients against sudden cardiac death (SCD), particularly in patients with VT and heart failure due to ischemic cardiomyopathy. However, inappropriate ICD therapy, especially inappropriate shock, is common in the real world. It has a significant morbidity rate and the potential to trigger ventricular arrhythmias, leading to cardiac decompensation and death. In the MADIT II experiment, these phenomena occurred in approximately 11.5% of patients.^[1]^ The most common cause of supraventricular tachycardia (SVT) is atrial fibrillation (44%), followed by SVT (36%). To our knowledge, this is the first case of inappropriate ICD therapy due to DAVNNRT in a patient with IHD and HFrEF in China. Many methods for minimizing inappropriate ICD therapy have been developed, such as the implantation of ICDs with an SVT-VT discrimination function, home monitoring, atrial sensing, and dual-chamber ICDs. However, in this case, the ICD identified DAVNNRT as VT because atrial sensing indicated a relationship between the atrium and ventricle as V > A, ultimately resulting in inappropriate ICD therapy.

Common types of NCT include sinus tachycardia, atrial fibrillation/flutter/tachycardia, atrioventricular node reentrant tachycardia (AVNRT), and atrioventricular reentrant tachycardia (AVRT). DAVNNRT is not a common type of NCT,^[2,3]^ and it was first described by Wu et al.^[4]^ The presence of imbalanced electrophysiological properties of the slow/fast pathway in the AVN usually generates AVNRT.^[5]^ In rare situations, supraventricular beats can occur simultaneously with fast and slow pathways, which generates 1:2 AV conduction and causes DAVNNRT. The decisive conditions for this arrhythmia are as follows: changes in sinus excitation, occurrence of atrial and ventricular premature beats, and the need for the electrophysiological characteristics (including conduction velocity, refractory period, and backward conduction) of the 2 pathways and the distal common pathway to differ. The middle panel of Figure 3d shows the tachycardia-building process. These conditions are affected by the autonomic nervous system and various drugs in most cases; therefore, they rarely occur in the real world. Peiker et al^[6]^ have accounted for the electrophysiological characteristics of DAVNNRT in a review of 68 cases from 1995 to 2014. The authors indicated that the most significant indication of DAVNNT on ECG is a P wave followed by 2 narrow QRS complexes. Because it is not widely known, DAVNNRT may be diagnosed as atrial fibrillation, atrial premature beats, or other SVTs, especially since dual AV nodal conduction may be intermittent.^[7,8]^ Bigeminal junctional ectopy could be another arrhythmogenic mechanism for this, but it usually shows more irregular variations in the coupling interval with the previous sinus beat.^[9]^ DAVNNRT has a fixed H-V interval and a slight variation in the R1R2 interval. Due to the variable conduction of the slow and fast pathways, slight changes in QRS morphology may be detected. As recently described by De Ponti et al,^[10]^ the different inputs into the bundle of His from the fast and slow pathways, suggesting the longitudinal dissociation of the distal AVN extending to the bundle of His (referred to as Zhang's^[11]^ phenomenon (or His electrogram alternans)), potentially explains the different QRS complex morphologies. Cardiovascular diseases, such as IHD, could cause changes in the extent and heterogeneity of structural discontinuities. The variability of cardiac cycle time and ventricular wall tension between the 2 ventricular beats may also contribute to the minimal difference between the 2 QRS complexes in this patient. The difference between 2 AH intervals ranged from 265 to 520 ms (359 ± 46 ms), suggesting the different electrophysiological characteristics of the 2 pathways, and the slow pathway conduction was slow enough to allow the His-Purkinje system to recover excitability after being depolarized by the first excitation over the fast pathway.^[6,12,13]^ The retrograde conduction between the ventricle and atrium was weak or absent, as has been observed in previous studies.^[6,8,10]^ As described by Rivner et al^[14]^ article, supraventricular beats can only conduct down the fast pathway, both the fast and slow AV nodal pathways, and the slow pathway only. Of note, as with this patient, atrial beats at a rate of 100 BPM are conducted along the slow pathway (Fig. 1e). In contrast, as shown in Figure 2b, atrial beats at a rate of 50 BPM are conducted along the fast pathway, which is probably due to the different electrophysiological characteristics of dual AVN pathways, the presence of concealed retrograde conduction, and the refractory period of the distal bundle of His. This finding is illustrated in the left and right panels of Figure 3d. Interestingly, tachycardia and inappropriate ICD therapy mainly occur at an atrial rate between 60 and 100 BPM.

It is certain that DAVNNRT can be cured by effective ablation of the slow pathway to suppress its forward conduction and has a good prognosis. In fact, the dual pathway of AVN is common, and it is not necessary to eliminate the slow pathway for all patients only if it generates tachycardia or for patients who undergo implantation of an ICD.

In conclusion, DAVNNRT is a rare type of tachycardia that appears as an irregular narrow QRS complex with 1:2 AV conduction, especially discriminated as VT by the SVT-VT discriminator of the ICD. We present a rare reason for inappropriate ICD therapy. It is important for us to have a full understanding of this arrhythmia to avoid misdiagnosis and incorrect treatment.

## Acknowledgment

We thank AJE (https://www.aje.cn) for its linguistic assistance during the preparation of this manuscript.

## Author contributions

**Clinical data collection and interpretation:** Chengming Ma, Shiyu Dai, and Xiaohong Yu.

**Data curation:** Chengming Ma, Shiyu Dai.

**Editing and revision:** Xiaomeng Yin and Yunlong Xia.

**Figure drafting**: Chengming Ma and Wenwen Li.

**Formal analysis:** Xiaohong Yu.

**Software:** Wenwen li.

**Supervision:** Xiaomeng Yin, Yunlong Xia.

**Writing – original draft:** Chengming Ma.

**Writing – review & editing:** Xiaomeng Yin, Yunlong Xia, Lianjun Gao.
